# Preparation and Performance Optimization of Lead–Zinc Tailing Sintered Bricks

**DOI:** 10.3390/ma18061381

**Published:** 2025-03-20

**Authors:** Dongliang He, Yanhui Cheng, Rui Li, Hang Lin

**Affiliations:** 1 School of Civil Engineering, Hunan City University, Yiyang 413000, China; hedongliang14@126.com (D.H.); chengyanhuihn@126.com (Y.C.); 2 School of Resources and Safety Engineering, Central South University, Changsha 410083, China; 215511001@csu.edu.cn

**Keywords:** lead–zinc tailings, high content, sintered bricks, compressive strength, SEM

## Abstract

Lead–zinc tailings are waste materials generated from mineral processing and smelting, and their long-term accumulation poses potential threats to the environment and soil. To achieve resource recycling and sustainable development, this study used lead–zinc tailings and clay as raw materials and glass powder as a modifier to prepare modified lead–zinc tailing sintered bricks. Through full-factor experiments and single-factor experiments, the effects of the material proportions, the sintering temperature, and the holding time on the properties of the sintered bricks were investigated. The results show that the addition of glass powder significantly enhanced the compressive strength of the sintered bricks, reduced their water absorption rate, and improved their volume shrinkage rate. The optimal preparation conditions were as follows: 9% glass powder content, 90% lead–zinc tailings content, a sintering temperature of 1060 °C, and a holding time of 60 min. The resulting sintered bricks met the MU30-strength-grade requirements of the national standard for ordinary sintered bricks (GB/T5101-2017). The sintering temperature has a significant impact on brick performance; the compressive strength first increases, and then decreases, the water absorption rate continues to decrease, and volume change shifts from expansion to contraction. The influence of holding time was relatively weaker, but as the holding time increased, the compressive strength and the water absorption rate of the sintered bricks gradually stabilized. XRD and SEM analyses indicated that the minerals in the lead–zinc tailings decomposed and recrystallized during the sintering process. The liquid phase melt from the glass powder filled the pores and enhanced skeletal strength, thereby improving the microstructure and properties of the sintered bricks. The research findings provide a theoretical basis and practical guidance for the efficient utilization and building material application of lead–zinc tailings.

## 1. Introduction

Lead–zinc tailings, the waste products generated after the mineral processing and smelting of lead–zinc ores, are a significant environmental and resource management concern [1]. These tailings contain valuable metals and minerals that are not fully extracted during the initial processing stages. The long-term accumulation of lead–zinc tailings poses substantial risks, including the potential to trigger geological disasters such as debris flows and landslides. Moreover, the heavy metal ions present in tailings, such as Pb^2+^ and Zn^2+^, can leach into soil and groundwater, leading to severe water and soil pollution [2,3,4]. Consequently, the comprehensive utilization and proper disposal of lead–zinc tailings have emerged as urgent issues that need to be addressed to achieve effective resource utilization and sustainable environmental development. The presence of useful minerals in the tailings after the initial flotation process presents an opportunity for further resource recovery [5,6,7]. Secondary flotation is a promising approach that can remove impurities from the tailings and enhance the grade and recovery rate of concentrates by optimizing the operating conditions and employing efficient flotation reagents. This process not only contributes to resource recovery and increased utilization, but also mitigates the negative environmental impacts associated with tailing accumulation. The current secondary flotation processes for lead–zinc tailings encompass a variety of techniques, including chemical leaching, microbial leaching, magnetic roasting–magnetic separation processes, and advanced flotation technologies. However, the secondary tailings left after secondary flotation, which are deemed not valuable for further flotation, still pose potential threats to the environment and soil when accumulated over long periods. Given that these tailings are rich in elements such as silicon and aluminum, which are essential for building materials, they represent an important raw material source for the production of building materials. Utilizing lead–zinc tailings to produce building materials can effectively consume a large amount of tailing waste, thereby reducing the environmental burden and generating economic benefits for enterprises. Therefore, it is feasible to consider using lead–zinc tailings that are not valuable for secondary flotation as raw materials for the production of building materials [8,9].

At present, a significant number of scholars and research institutions have devoted themselves to exploring the preparation of building materials from tailings and have achieved remarkable outcomes [10,11,12,13,14]. These building materials encompass tailing bricks [15,16,17,18], foam ceramics [19,20,21,22], and ultra-high-performance concrete [23,24,25], which are extensively applied in various fields such as construction and road building. Wang et al. [26] investigated the influence of sintering temperature on the leaching behavior of heavy metal ions in lead–zinc tailings bricks and revealed that heavy metal ions can be effectively immobilized when the sintering temperature surpasses 1050 °C. Behera et al. [27,28] carried out research on the preparation of mine backfill paste utilizing lead–zinc tailings. Guo et al. [29] employed tungsten tailings to manufacture ceramic permeable bricks. Kang et al. [30] produced eco-friendly bricks using slate tailings and scrutinized their synthesis and characteristics. Kim et al. [31] prepared porous bricks incorporating red mud and tailings. Li et al. [32] explored the feasibility of fabricating low-cost glass ceramics by combining urban waste and lead–zinc tailings. Lin et al. [33] successfully prepared lead–zinc tailing bricks with a strength exceeding 30 MPa using lead–zinc tailings, fly ash, and clay as raw materials and determined the optimal process parameters to be a ratio of lead–zinc tailings/clay/fly ash = 6:3:1, a forming pressure of 20 MPa, a firing temperature of 1080 °C, and a holding time of 60 min. However, despite extensive research on utilizing lead–zinc tailings in building materials, several limitations are evident in the current literature. Most studies have focused on specific applications, such as mine backfill paste [27,28] or specialized bricks using tungsten and slate tailings [29,30], which may not be directly applicable to the production of general building bricks. While some researchers have achieved the effective immobilization of heavy metal ions at high sintering temperatures [26] or fabricated high-strength bricks [33], these methods often involve complex raw material ratios and high firing temperatures, which can increase production costs and energy consumption. Additionally, the introduction of additional materials, such as red mud [31] and urban waste [32], to enhance product properties may complicate the recycling process and limit the utilization rate of lead–zinc tailings. Overall, these studies have not fully optimized the utilization rate of lead–zinc tailings for the cost-effective and large-scale production of building materials.

Enhancing the utilization rate of lead–zinc tailings holds substantial theoretical and engineering application value for reducing tailing accumulation, lowering building material costs, and achieving resource recycling. This study aims to increase the utilization rate of lead–zinc tailings by preparing modified lead–zinc tailing sintered bricks using lead–zinc tailings and clay as raw materials and glass powder as a modifier. The impact of material proportions is examined through a full-factor experiment, and the optimal sintering temperature and holding time are identified through single-factor experiments. The sintering mechanism of the modified lead–zinc tailing sintered bricks is elucidated using XRD and SEM techniques. Sintering temperature has a significant impact on brick performance; compressive strength first increases, and then decreases, the water absorption rate continues to decrease, and volume change shifts from expansion to contraction. This comprehensive approach is expected to provide a more efficient and cost-effective solution for utilizing lead–zinc tailings in building materials.

## 2. Experiment

### 2.1. Experimental Materials

**Figure 1:**
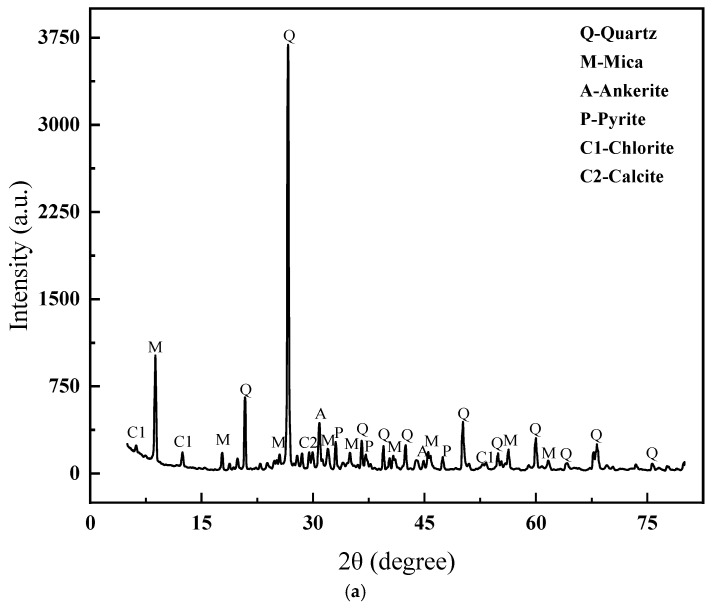

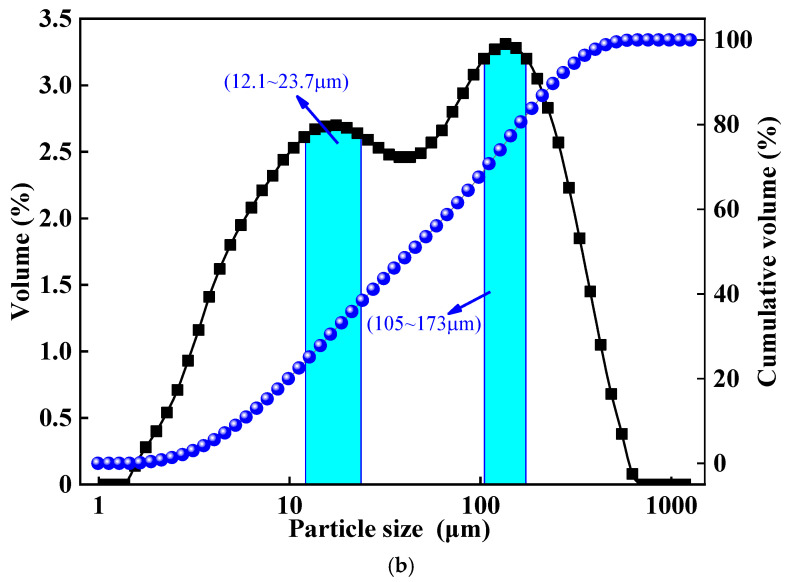
XRD patterns and particle size distribution of lead–zinc tailings. (**a**) XRD patterns distribution of lead–zinc tailings. (**b**) Particle size distribution of lead–zinc tailings.

This experiment utilized lead–zinc tailings and clay as the primary raw materials. The main chemical compositions of these raw materials were determined using X-ray fluorescence (XRF), as detailed in Table 1. The XRD patterns and particle size distribution of the lead–zinc tailings are presented in Figure 1. As can be observed, the mineral components of the lead–zinc tailings consist of quartz, mica, siderite, pyrite, chlorite, and calcite. Among these, quartz and mica are particularly abundant, characterized by high diffraction peak intensities and distinct crystalline features.

### 2.2. Modified Materials

The modifying agent employed in this study was glass powder, whose primary chemical constituents include SiO_2_, CaO, and Na_2_O. Glass powder does not exhibit distinct mineral crystal diffraction peaks. Instead, a broad peak is observed within the range of 20–30°, indicating that the main phase composition of glass powder is amorphous glass.

### 2.3. Experimental Scheme

#### 2.3.1. Specimen Preparation

The fired brick specimens were formulated using lead–zinc tailings, clay, and glass powder. The combined mass of lead–zinc tailings and clay was maintained at a constant level of 180 g. The dosage of glass powder was defined as the ratio of its mass to the total mass of lead–zinc tailings and clay. To explore the effects of glass powder dosage and tailings dosage on the properties of fired bricks, glass powder was added at four concentrations: 0%, 3%, 6%, and 9%. The dosage of lead–zinc tailings was set at seven concentrations: 70%, 75%, 80%, 85%, 90%, 95%, and 100%. A full factorial experiment was conducted, resulting in 28 different raw material ratios. The sintering parameters were set at a sintering temperature of 1060 °C and a holding time of 60 min. Additionally, based on the ratios that met the strength requirements, single-factor experiments were performed to investigate the effects of sintering temperature and holding time on the properties of lead–zinc tailing fired bricks. In the sintering temperature experiment, the holding time was kept constant at 60 min, while the sintering temperature was varied at five levels: 1020 °C, 1040 °C, 1060 °C, 1080 °C, and 1100 °C. In the holding time single-factor experiment, the sintering temperature was maintained at 1060 °C, and the holding time was varied at five durations: 20 min, 40 min, 60 min, 80 min, and 100 min. The specific preparation parameters for each fired brick specimen in this study are presented in Table 2.

**Figure 2:**
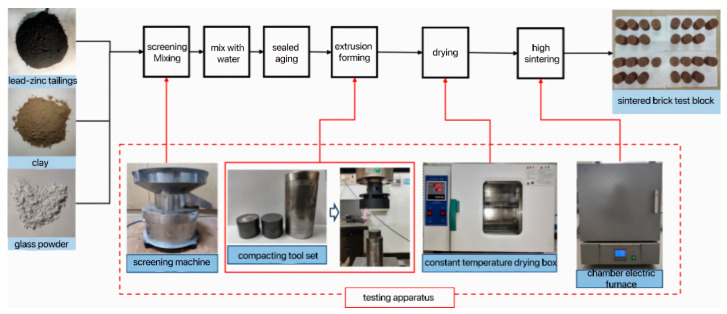
Preparation process of sintered bricks.

The preparation process of the sintered brick specimens was meticulously conducted as follows: Initially, the dried lead–zinc tailings, clay, and glass powder were thoroughly sieved through a 65-mesh screen to ensure a uniform particle size. Subsequently, these raw materials were precisely mixed in accordance with the predetermined ratios, and 12.5% water was carefully added to the mixture, which was then vigorously stirred to achieve homogeneity. The well-mixed material was subsequently placed into a sealed bag and subjected to aging at room temperature for a duration of 24 h. Following the aging process, the material was carefully poured into a steel mold with a diameter of Φ50 mm and a height of 50 mm, and pressed at a pressure of 20 MPa to form the specimen. The formed block was initially dried, and then meticulously placed into a box-type resistance furnace for sintering, ultimately yielding the sintered brick specimen. The detailed preparation process of sintered brick specimens is visually depicted in Figure 2.

#### 2.3.2. Parameter Determination

In accordance with the “Test Methods for Masonry Bricks” (GB/T 2542-2012), the primary performance indicators of the lead–zinc tailing sintered bricks were evaluated, encompassing compressive strength, the water absorption rate, and the volume shrinkage rate. To ensure the reliability and reproducibility of the results, each experimental condition was replicated three times. The average values and standard deviations of the test results were calculated to assess the quality and consistency of measurements. A compressive strength test was performed using the Hualong WHY-300 press (Shanghai Hualong Test Instrument Co., Ltd., Shanghai, China). Specifically, the specimen was precisely positioned on the loading platform, with the loading rate meticulously set at 0.4 MPa/s. The compressive strength of the sintered brick specimen was subsequently calculated based on the test results. The average compressive strength values and their corresponding standard deviations were found to be within an acceptable range, indicating the good reproducibility and precision of the testing process. The volume shrinkage rate was determined by measuring the dimensions of the specimens before and after sintering. As stipulated by “Sintered Common Bricks” (GB/T 5101-2017), the strength grades of the sintered bricks were categorized into M10, M15, M20, M25, and M30, corresponding to minimum compressive strengths of 10 MPa, 15 MPa, 20 MPa, 25 MPa, and 30 MPa, respectively. Additionally, the water absorption rate of the sintered bricks is required to be no more than 18%.

## 3. Experimental Results

### 3.1. Influence of Material Proportion on the Performance of Lead–Zinc Tailing Sintered Bricks

**Figure 3:**
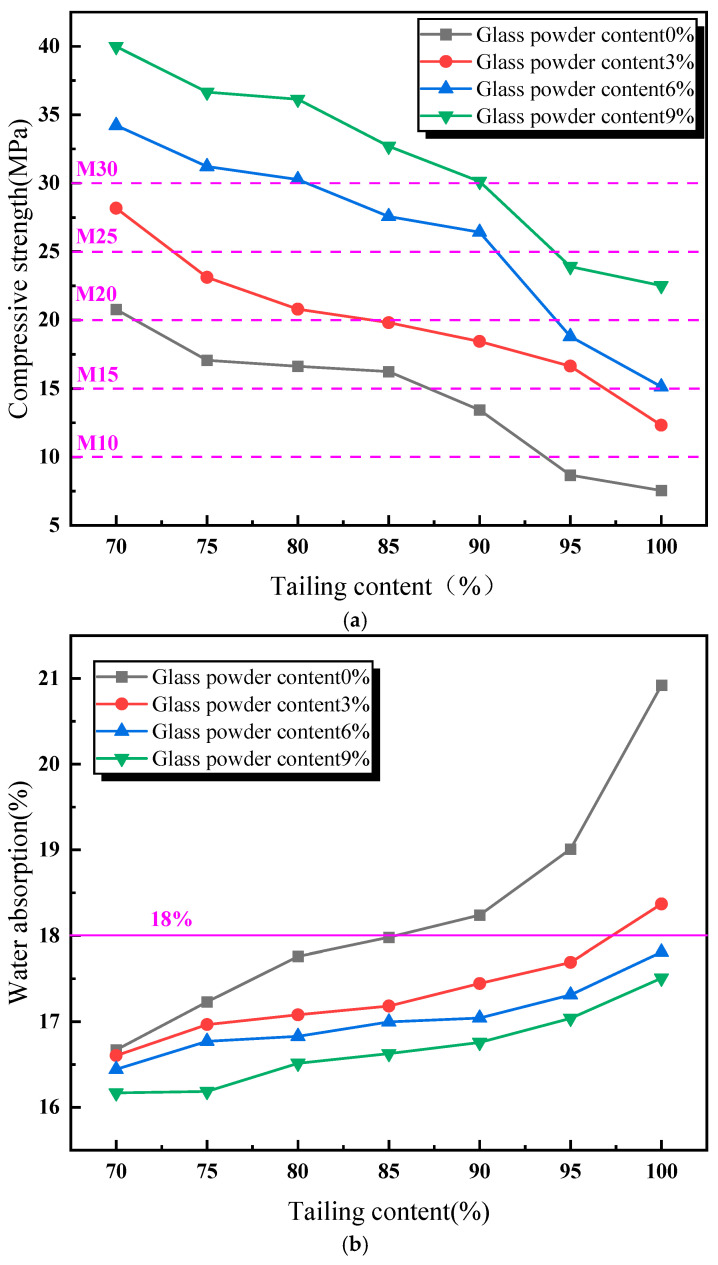

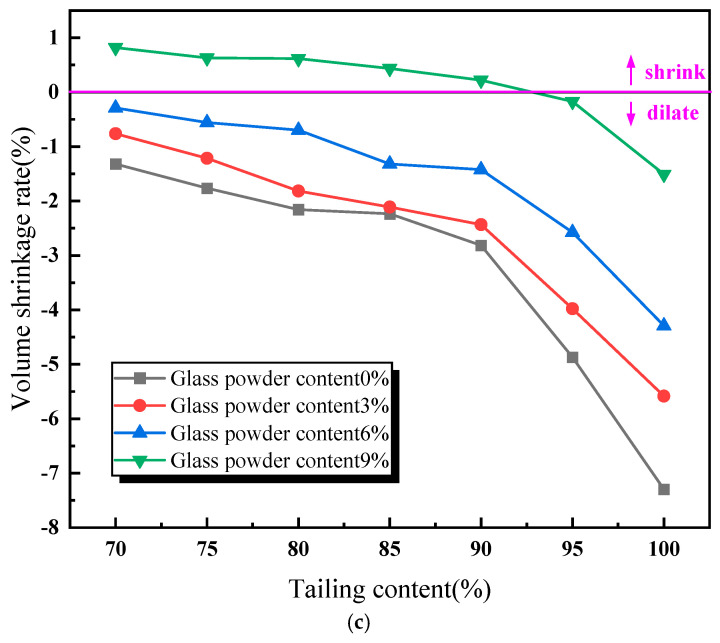
Influence of material proportion on performance of lead–zinc tailing sintered bricks. (**a**) Influence of material proportion on compressive strength. (**b**) Influence of material proportion on water absorption. (**c**) Influence of material proportion on volume shrinkage rate.

Figure 3 presents the influence of the addition of glass powder and tailing content on the compressive strength, the water absorption rate, and the volume shrinkage rate of the sintered bricks. As shown in Figure 3a, the compressive strength of the sintered bricks decreases with an increase in the content of lead–zinc tailings. The addition of glass powder has a substantial impact on improving the strength of the sintered bricks. Without the addition of glass powder, the maximum compressive strength of the sintered bricks is 20.8 MPa, which only meets the requirements of M20-grade sintered bricks, with a lead–zinc tailing content of 70%. When the glass powder content is 3%, the compressive strength of the sintered bricks with a lead–zinc tailing content of 80% still meets the M20-grade requirements. When the glass powder content reaches 6%, the compressive strength of the sintered bricks with a lead–zinc tailing content of 90% meets the M20-grade requirements. Compared with the sintered bricks without glass powder, when the glass powder content is 9%, the compressive strength of the sintered bricks with a lead–zinc tailing content of 100% meets the M20-grade requirements, and the compressive strength of the sintered bricks with a lead–zinc tailing content of 90% reaches 30.3 MPa, which meets the M30-grade strength requirements. During the high-temperature sintering process, the glass powder melts, and the generated liquid phase fills the internal voids of the sintered bricks, binding the tailings particles together, thereby enhancing the compressive strength of the sintered bricks. Moreover, the liquid melt formed significantly reduced the porosity of the sintered bricks after filling the voids.

According to Figure 3b, the water absorption rate of the sintered bricks increases with an increase in the content of lead–zinc tailings. Without the addition of glass powder, the increase in lead–zinc tailing content has a significant impact on the water absorption rate of sintered bricks. When the tailing content reaches above 85%, the water absorption rate of the sintered bricks exceeds 18%. The addition of glass powder has a significant effect on reducing the water absorption rate of the sintered bricks. When the glass powder content reaches 3%, only the water absorption rate of the sintered bricks with a lead–zinc tailing content of 100% exceeds 18%. When the glass powder content reaches 6% or more, the water absorption rate of the sintered bricks meets the standard requirements.

As illustrated in Figure 3c, the volume shrinkage rate of the lead–zinc tailing sintered bricks decreases with an increase in the content of lead–zinc tailings. When the glass powder content is between 0% and 6%, the volume shrinkage rate of the sintered bricks is negative, indicating that the specimens expanded in volume during the high-temperature sintering process. When the glass powder content reaches 9%, the volume shrinkage rate of sintered bricks ranges from −1.51% to 0.82%. When the lead–zinc tailing content does not exceed 90%, the volume shrinkage rate of the sintered bricks is positive. Moreover, it can be observed from the trends of the curves in Figure 3 that with an increase in glass powder content, the trend of the compressive strength curve decreasing with an increase in tailing content remains almost unchanged, while the trends of the water absorption rate and the volume shrinkage rate curves are significantly reduced.

In summary, the two groups of specimens with a “6% glass powder content and 80% lead–zinc tailing content” and a “9% glass powder content and 90% lead–zinc tailing content” meet the M30 requirements in terms of compressive strength, the water absorption rate, and the volume shrinkage rate. These two groups of specimens were selected for the analysis of the influence of sintering parameters on the performance of the lead–zinc tailing sintered bricks.

### 3.2. Influence of Sintering Temperature on the Performance of Lead–Zinc Tailing Sintered Bricks

**Figure 4:**
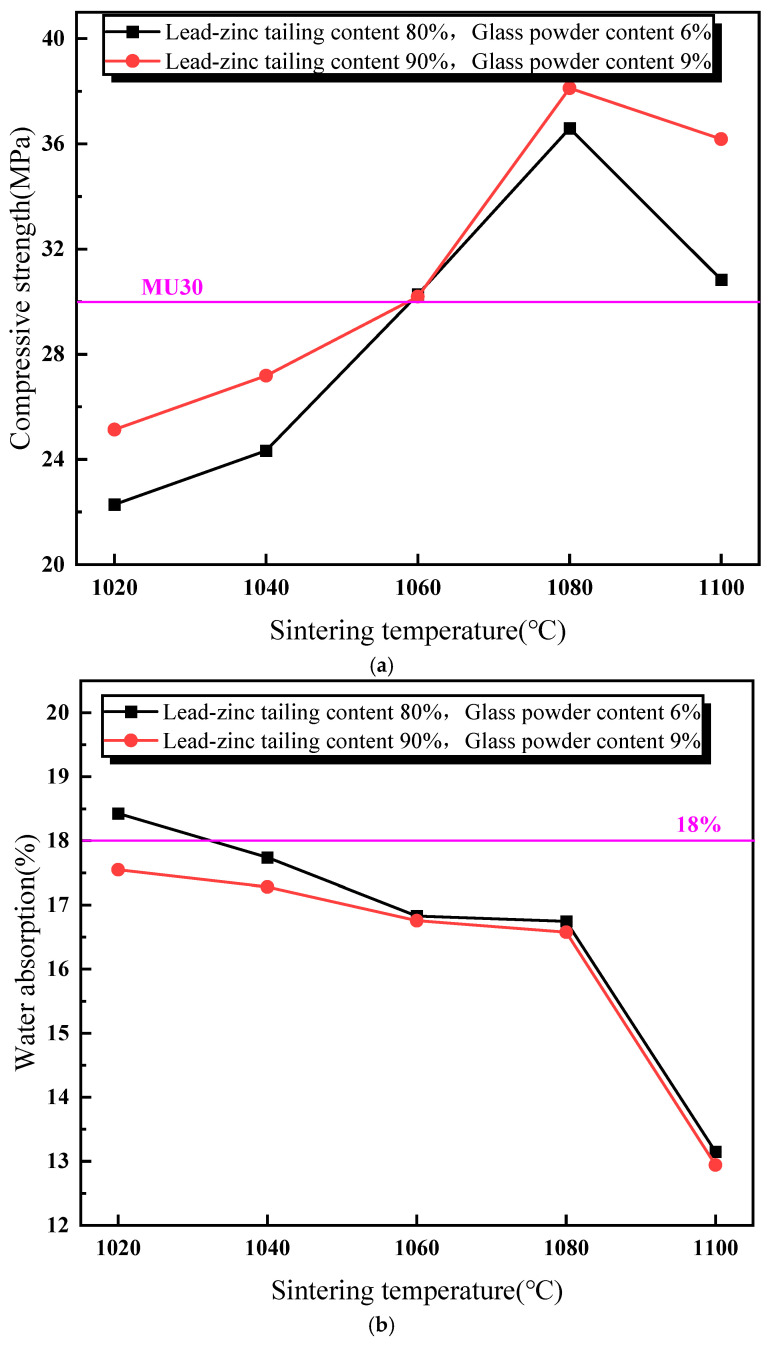

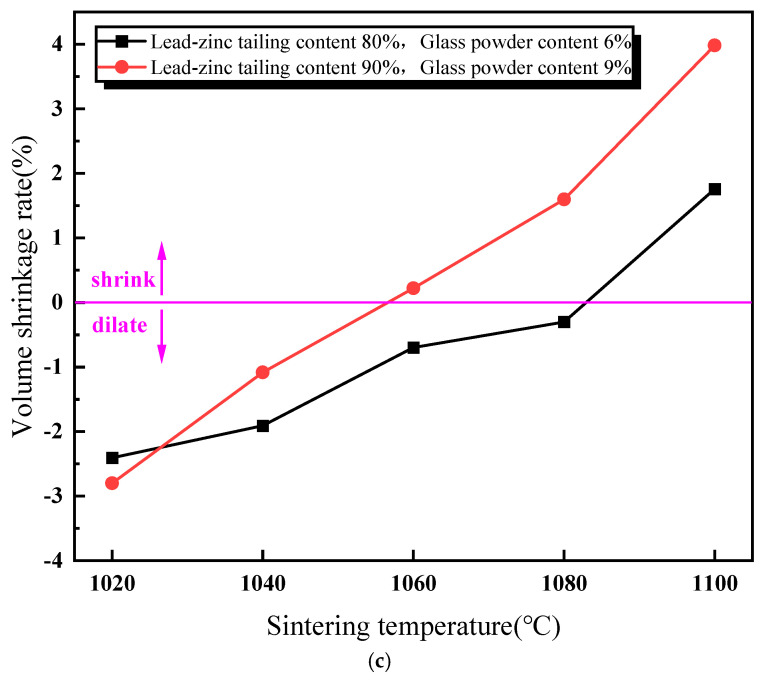
Influence of sintering temperature on properties of lead–zinc tailing sintered bricks. (**a**) Influence of sintering temperature on compressive strength. (**b**) Influence of sintering temperature on water absorption. (**c**) Influence of sintering temperature on volume shrinkage rate.

Figure 4 illustrates the impact of sintering temperature on the performance of the sintered brick specimens. As shown in Figure 4a, the compressive strength of the sintered bricks initially increases, and then decreases with the rise in sintering temperature. When the sintering temperature exceeds 1060 °C, the compressive strength of the bricks meets the M30-strength-grade requirement for both the material mixtures. The maximum compressive strength is achieved at a sintering temperature of 1080 °C. Figure 4b depicts the relationship between the water absorption rate and sintering temperature for the two material mixtures. It is observed that the water absorption rate of the sintered bricks decreases gradually with an increasing sintering temperature. At a sintering temperature of 1100 °C, a significant reduction in the water absorption rate is noted for both the mixtures. Figure 4c shows the variation in volume shrinkage rate of the sintered bricks with an increasing sintering temperature. The volume shrinkage rate is positively correlated with the sintering temperature. At a sintering temperature of 1060 °C, the volume shrinkage rate of the bricks with 9% glass powder and 90% lead–zinc tailings turns positive. For the bricks with 6% glass powder and 80% lead–zinc tailings, the volume shrinkage rate becomes positive only at 1100 °C. The increase in sintering temperature promotes the reactions of the mineral components within the lead–zinc tailings and the melting of glass powder to form the liquid phase. The liquid phase, with its surface tension, binds the surrounding particles together, enhancing the compressive strength and reducing volume expansion. Additionally, the liquid phase flows into the surrounding pores under gravity, thereby reducing the water absorption rate.

### 3.3. Influence of Soaking Time on the Properties of Lead–Zinc Tailing Sintered Bricks

**Figure 5:**
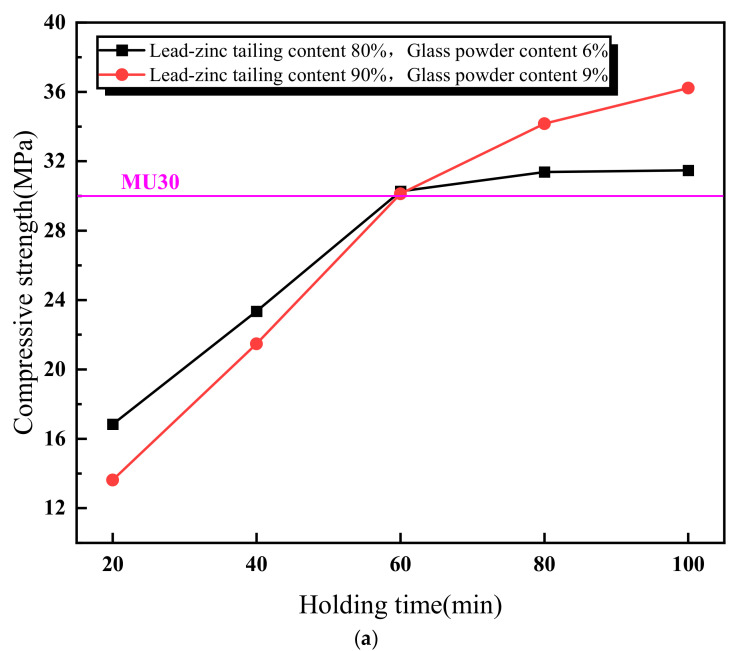

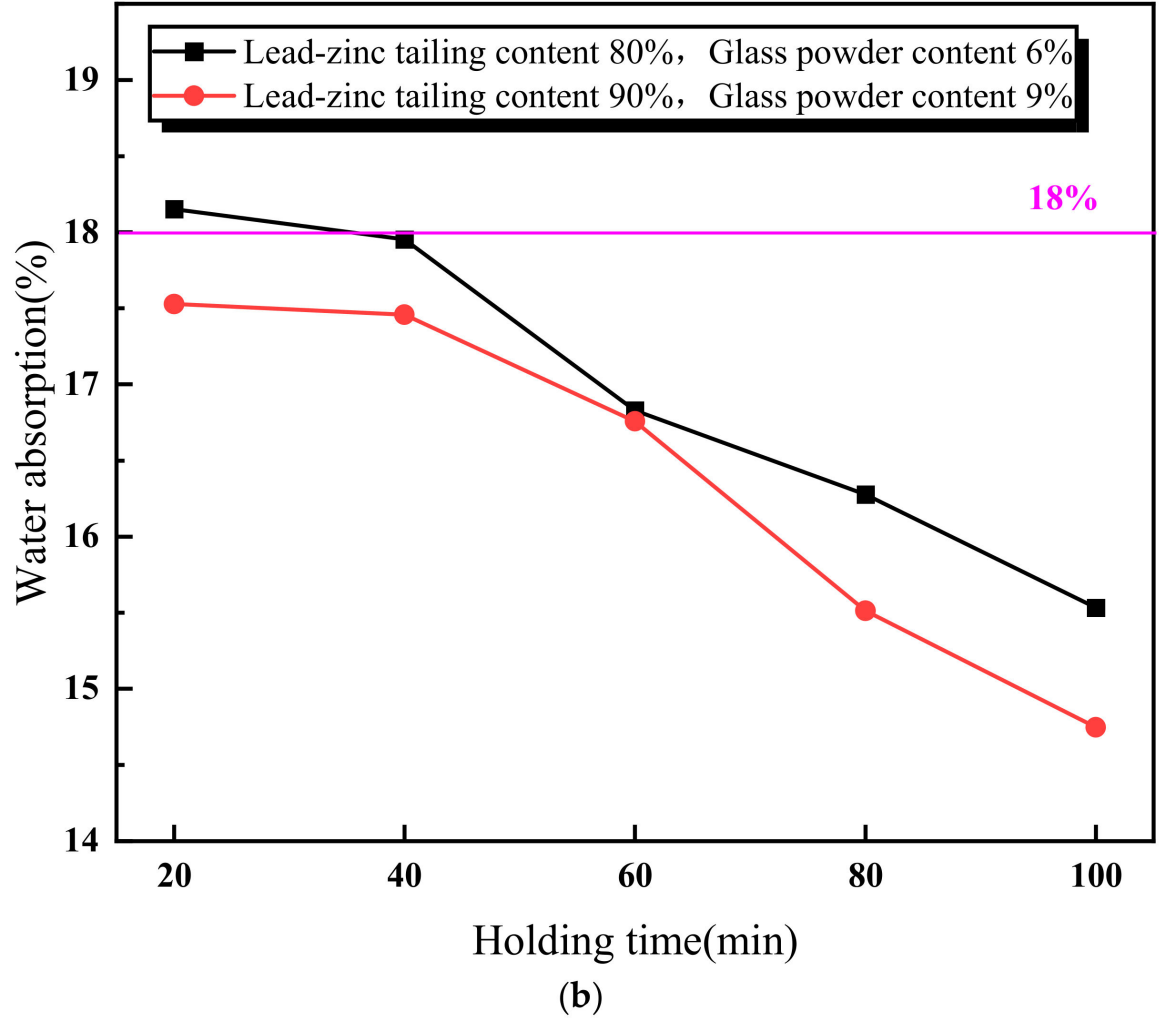

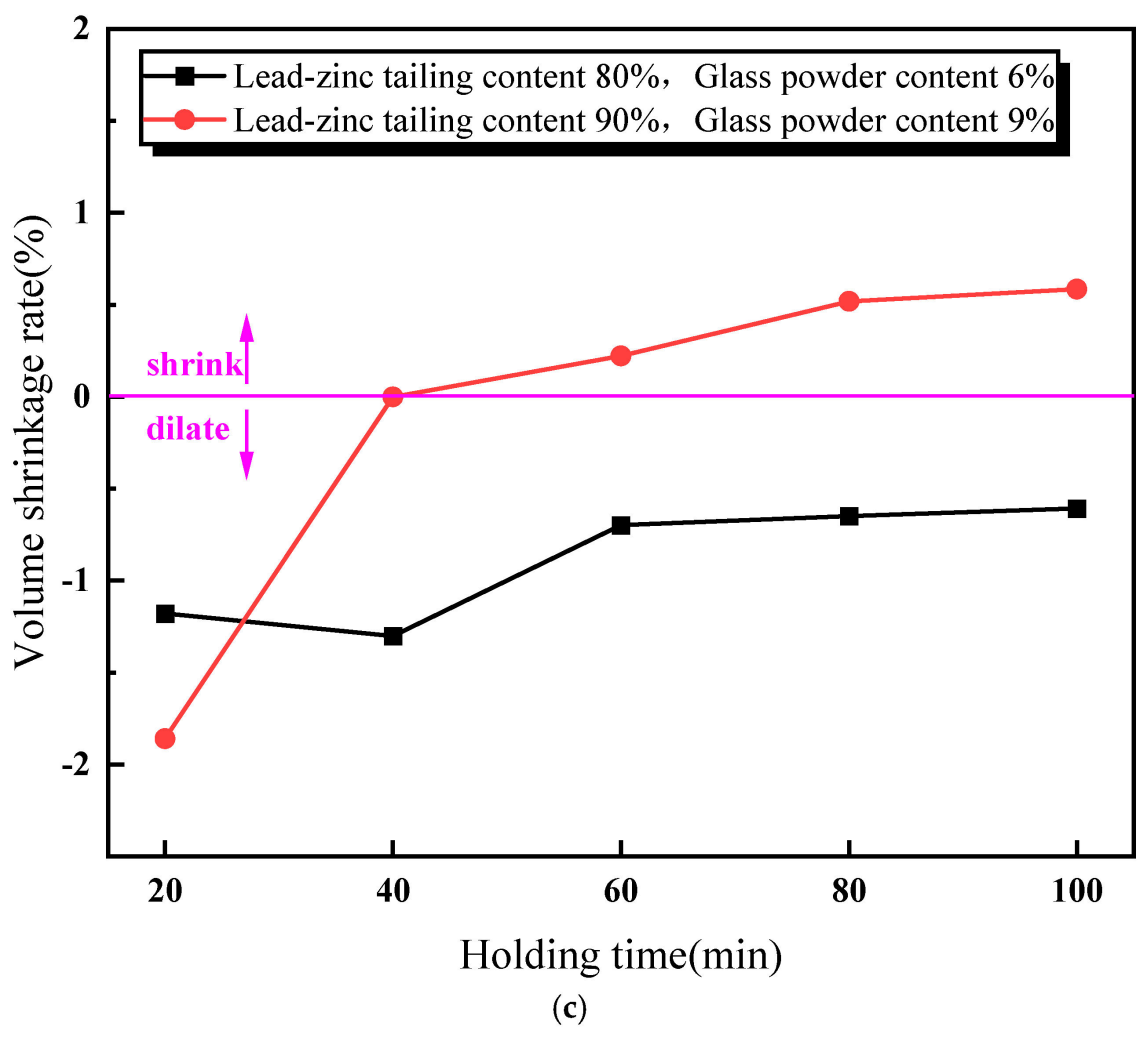
Influence of soaking time on properties of lead–zinc tailing sintered bricks. (**a**) Influence of soaking time on compressive strength. (**b**) Influence of soaking time on water absorption. (**c**) Influence of soaking time on volume shrinkage rate.

Figure 5 illustrates the relationship between the properties of the lead–zinc tailing sintered bricks and the soaking time. As shown in Figure 5a, the compressive strength of the sintered bricks increases with the increase in soaking time. When the soaking time exceeds 60 min, the compressive strength meets the M30-strength-grade requirement. Additionally, the rate of increase in compressive strength slows down when the soaking time is above 60 min. Figure 5b shows the relationship between the water absorption rate of the sintered bricks and soaking time. The water absorption rate decreases continuously with increasing soaking time. When the soaking time reaches 40 min, the water absorption rate of the sintered bricks with both the material mixtures meets the required standard. Figure 5c indicates that the effect of soaking time on the volume shrinkage rate of the sintered bricks with 6% glass powder and 80% lead–zinc tailings is relatively weak. The volume shrinkage rate of these bricks fluctuates slightly upward with increasing soaking time and remains negative, indicating that volume expansion is the dominant factor. For the sintered bricks with 9% glass powder and 90% lead–zinc tailings, the volume shrinkage rate increases from −1.86% to −0.01% when the soaking time increases from 20 min to 40 min. When the soaking time reaches 60 min and above, the volume shrinkage rate becomes positive.

## 4. Sintering Mechanism Analysis

### 4.1. Mechanism of Experimental Materials on Lead–Zinc Tailing Sintered Bricks

**Figure 6:**
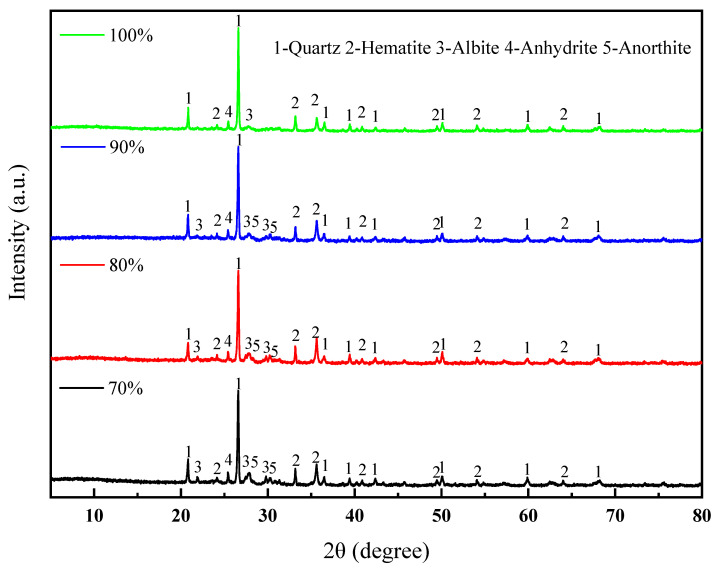
XRD patterns of lead–zinc tailing sintered bricks with different lead–zinc tailing contents.

Under the conditions of a forming pressure of 20 MPa, a sintering temperature of 1060 °C, and a soaking time of 60 min, the XRD patterns of the sintered bricks with different lead–zinc tailing contents are shown in Figure 6. With an increase in the amount of lead–zinc tailings added, the diffraction peaks of feldspar minerals gradually disappear, indicating that the content of feldspar minerals in the sintered bricks is decreasing. There are two main reasons for this. On the one hand, some of the feldspar originates from clay. As the amount of lead–zinc tailings added increases, the clay content in the sintered bricks decreases, which in turn leads to a reduction in the feldspar content. On the other hand, the formation of feldspar requires the participation of oxides such as SiO_2_ and Al_2_O_3_, which are present in relatively low amounts in lead–zinc tailings. The less feldspar is generated in the solid-phase reaction, the less feldspar will precipitate during the cooling process, resulting in a weaker diffraction intensity of feldspar. Sodium feldspar and calcium feldspar are very strong and can also promote the formation of a liquid phase, which can enhance the mechanical properties of sintered bricks. However, the addition of lead–zinc tailings is not conducive to the formation of feldspar. Therefore, macroscopically, it cannot provide sufficient mechanical strength for sintered bricks, leading to a gradual decrease in the strength of the sintered bricks with the increasing amount of lead–zinc tailings added.

**Figure 7:**
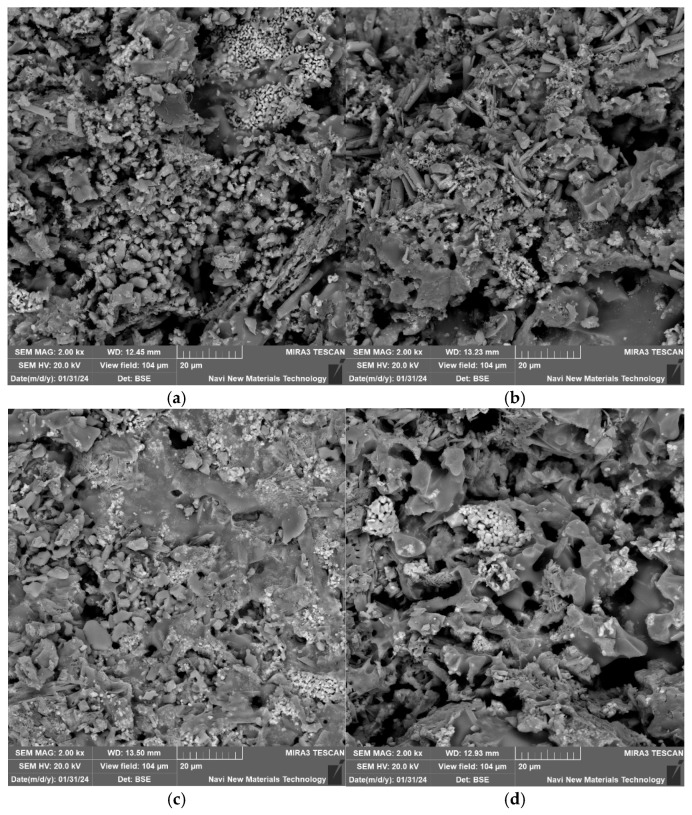
SEM analysis of sintered bricks with different glass powder contents (2000×). (**a**) Glass powder content 0%. (**b**) Glass powder content 3%. (**c**) Glass powder content 6%. (**d**) Glass powder content 9%.

Under the conditions of a forming pressure of 20 MPa, a sintering temperature of 1060 °C, a lead–zinc tailing content of 90%, and a soaking time of 60 min, the microstructures of the lead–zinc tailing sintered bricks with different glass powder contents are shown in Figure 7. When the glass powder content is 0%, the glassy substances in the sintered bricks exist primarily in the form of microspheres, and the overall structure is relatively loose, with a large number of micropores present, some of which are interconnected. When the glass powder content is 3% and 6%, extensive glassy substances begin to appear, and the number of pores gradually decreases, indicating that the addition of glass powder promotes an increase in the amount of melt within the sintered bricks. When the glass powder content reaches 9%, more blocky, irregular glassy substances and surrounding crystalline structures can be observed, and the cross-section of the sintered bricks becomes more compact. These glassy substances are the products of the cooling of the molten liquid phase, and they interconnect with each other, effectively isolating the ingress of water. The addition of glass powder is more conducive to the formation and distribution of these glass phases, which can transform the skeletal structure of the bricks from “loose” to “monolithic”, thereby enhancing the cohesion of the specimens and strengthening their resistance to destruction. Macroscopically, this contributes to the improvement of the compressive strength and elastic modulus of the sintered bricks.

### 4.2. Mechanism of Sintering Temperature on Lead–Zinc Tailing Sintered Bricks

**Figure 8:**
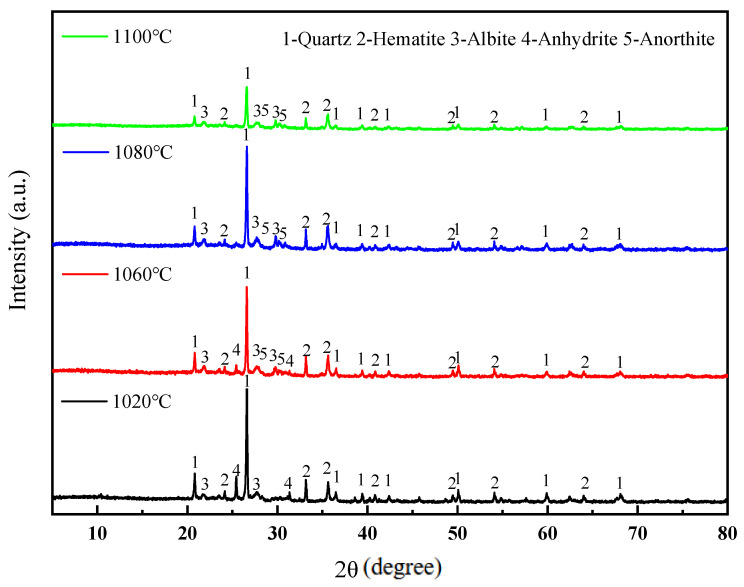
XRD patterns of lead–zinc tailing sintered bricks at different sintering temperatures.

Figure 8 presents the XRD patterns of the sintered bricks prepared at different sintering temperatures. The phase changes in the sintered bricks at different sintering temperatures can be observed. Firstly, the diffraction peaks of feldspar minerals are enhanced, and the diffraction peaks of anorthite begin to appear at 1060 °C. The formation of anorthite requires the participation of CaO. As the temperature increases, the decomposition reactions of minerals such as calcite and siderite become more complete, providing conditions for the solid-phase reaction to generate anorthite. Meanwhile, the increase in temperature also promotes the growth and development of feldspar mineral crystals. When the sintering temperature is further increased, feldspar crystals begin to develop. The accelerated formation of feldspar speeds up the generation of the liquid phase, which is macroscopically manifested as an increase in glassy substances and the enhancement of the strength of the sintered bricks. Secondly, Figure 8 also shows that the diffraction intensity of quartz gradually weakens with the increase in temperature, especially for the green brick bodies sintered at 1100 °C, where the diffraction intensity of quartz becomes even weaker. This is because quartz is easily melted at high temperatures, especially in the presence of feldspar, where quartz can form low-melting eutectics with feldspar and other aluminosilicates. This molten liquid phase can further dissolve the remaining quartz, and ultimately, upon cooling, form amorphous glassy substances, leading to changes in the crystalline structure of quartz and a reduction in the intensity of its diffraction peaks. The strength of the sintered bricks mainly comes from the skeleton structure composed of mineral crystals such as quartz and feldspar. The glassy substances generated after sintering can bond quartz and feldspar, increasing the density and further enhancing the rigidity of the skeleton. However, the glass phase itself has relatively low strength. When the sintering temperature is too high, resulting in a reduction in quartz and an increase in the glass phase, it is not conducive to the development of the mechanical properties of the sintered bricks. Macroscopically, this is manifested as a decrease in the compressive strength and elastic modulus of the lead–zinc tailing sintered bricks when the sintering temperature is raised to 1100 °C.

**Figure 9:**
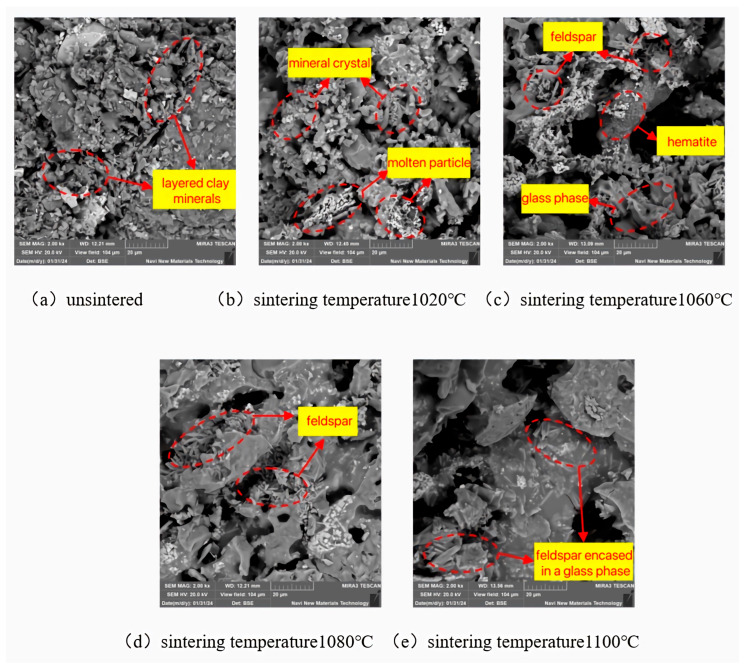
SEM characteristics of lead–zinc tailing sintered bricks at different sintering temperatures.

As depicted in Figure 9, the particles of the unsinistered specimens have distinct edges and corners, with the layered clay minerals being more pronounced, and the contact between particles is relatively loose. When the sintering temperature is 1020 °C, the edges and corners of some particles are almost eliminated, and the surfaces become slightly spherical. Meanwhile, the formation of mineral crystals and the appearance of numerous micropores between particles can be observed. This is because under the influence of a high temperature, the particle surfaces undergo melting, forming spherical glassy phases. At the same time, some substances disappear to form pores, and new mineral crystals are generated. As the sintering temperature increases, the development of feldspar crystals accelerates, and the glass powder further melts, leading to an increase in the high-temperature molten liquid phase. Figure 9c,d shows that the long-columnar feldspar crystals gradually increase in number, and the distribution of glassy substances expands. Feldspar, quartz, and the glassy phase are closely integrated, forming a “monolithic” dense structure. This structure can enhance the attraction between particles and resist external disturbances. Macroscopically, it is manifested as an improvement in the strength properties of the sintered bricks and a reduction in water absorption. When the sintering temperature is 1100 °C, the surface of the specimen becomes smooth and flat, and the mineral crystals are almost entirely enveloped by the glassy substances. The number of feldspar crystals decreases, and their crystalline structure is no longer distinct. This indicates that at a sintering temperature of 1100 °C, the glass powder melts more rapidly, and more quartz and feldspar participate in the melting reaction, generating a large amount of liquid phase material. These liquid phase materials envelop the crystalline structures, significantly reducing the number of pores and weakening the supporting role of the particle framework. Macroscopically, this is manifested as a weakening of the strength properties of the sintered bricks.

### 4.3. Mechanism of Soaking Time on Lead–Zinc Tailing Sintered Bricks

**Figure 10:**
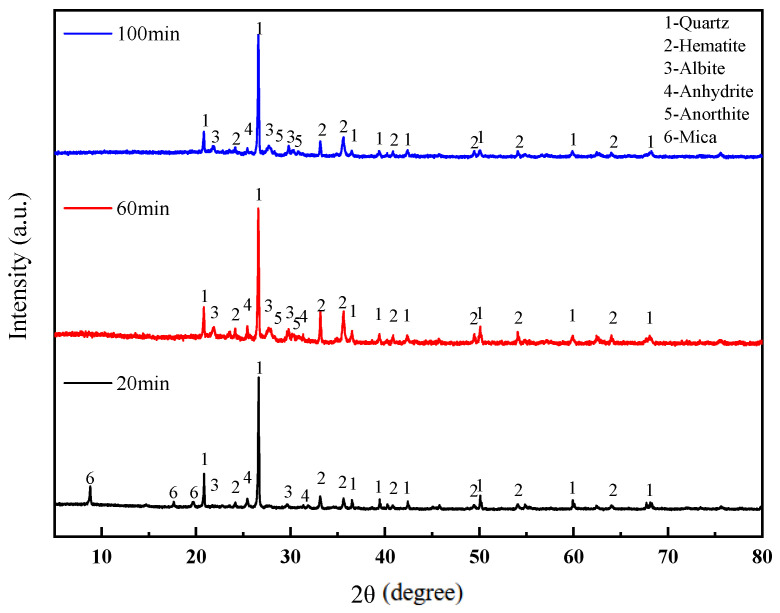
XRD patterns of lead–zinc tailing sintered bricks at different soaking times.

The XRD patterns of the sintered bricks at different soaking times is shown in Figure 10. It can be observed that when the soaking time is 20 min, mica minerals are still present in the sintered bricks, indicating that the decomposition of some minerals had fully completed due to the insufficient soaking time. As the soaking time is extended, the amount of mica gradually decreases, while the amount of feldspar minerals increases. This is because the decomposition of mica can generate SiO_2_ and Al_2_O_3_, which are essential components for the formation of feldspar. The liquid phase in the system interacts with the remaining solid particles and crystals, promoting a more compact structure. Therefore, as the soaking time increases, the compressive strength of the sintered bricks continuously improves, and their permeability gradually decreases.

**Figure 11:**
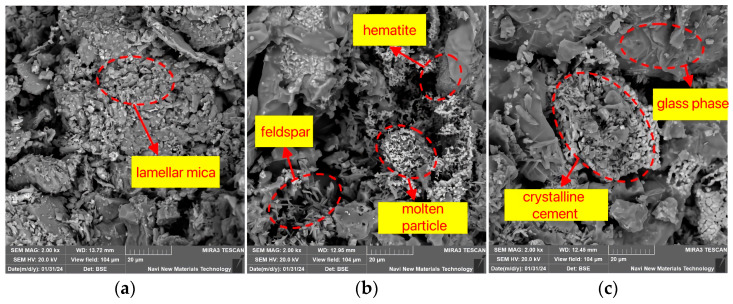
SEM characteristics of lead–zinc tailing sintered bricks at different soaking times. (**a**) Holding time 20 min. (**b**) Holding time 60 min. (**c**) Holding time 100 min.

Figure 11 illustrates the effect of soaking time on the internal microstructure of the sintered bricks. When the soaking time is 20 min, unreacted mica particles with a layered structure can be observed, and the distribution of newly formed mineral crystals and glassy substances is sparse. At this point, the contact between the sample particles is relatively loose, and the skeletal structure is not dense enough. As the soaking time increases, Figure 11b clearly shows that the sample particles exhibit melting phenomena, with an increased distribution of feldspar mineral crystals and the appearance of large areas of glassy substances and mineral crystallization. Subsequently, the liquid phase further increases in quantity, with a more uniform and extensive distribution, ultimately forming blocky glass phases and dense crystalline bonds, as shown in Figure 11c. Macroscopically, this enhances the strength properties of the sintered bricks.

## 5. Conclusions

(1) This paper provides insights into the preparation and performance optimization of lead–zinc tailing sintered bricks. The addition of glass powder significantly enhances the compressive strength, reduces the water absorption rate, and improves the volume shrinkage rate of the sintered bricks. The optimal preparation conditions are identified as a 9% glass powder content, a 90% lead–zinc tailing content, a sintering temperature of 1060 °C, and a soaking time of 60 min.
(2) The sintering temperature plays a crucial role in determining the properties of the sintered bricks. As the sintering temperature increases, compressive strength initially rises, and then declines, while the water absorption rate continues to decrease. Volume change shifts from expansion to contraction with an increasing sintering temperature. Although the effect of soaking time is relatively weaker, it still has a notable impact on the properties of the sintered bricks. With prolonged soaking time, the compressive strength and water absorption rate of the sintered bricks gradually stabilize.
(3) The sintering process induces the decomposition and recrystallization of minerals in the lead–zinc tailings. The liquid phase melt from the glass powder fills the pores and strengthens the skeletal structure, thereby improving the microstructure and properties of the sintered bricks. This research offers a theoretical basis and practical guidance for the efficient utilization of lead–zinc tailings in building materials, contributing to resource recycling and sustainable development.

## Figures and Tables

**Table 1:** Major chemical composition of experimental materials (unit: %).

Material	SiO_2_	Al_2_O_3_	Fe_2_O_3_	MgO	CaO	K_2_O	Na_2_O	MnO_2_	TiO_2_	ZnO	Other
Tailings	48.17	10.79	14.15	4.14	4.20	3.01	0.456	0.73	0.312	0.493	13.55
Clay	61.37	14.32	4.74	2.36	12.40	2.59	1.03	/	/	/	1.19

**Table 2:** Parameters of lead–zinc tailing sintered bricks.

Experimental Variables	Experimental Category	Lead–Zinc Tailing Content (%)	Glass Powder Content (%)	Sintering Temperature (°C)	Soaking Time (min)
Material Content	Full Factorial Experiment	70, 75, 80, 85, 90, 95 and 100	0, 3, 6 and 9	1060	60
Sintering Temperature	Single-Factor Experiment	90	9	1020, 1040, 1060, 1080 and 1100	60
Soaking Time	Single-Factor Experiment	90	9	1060	20, 40, 60 80 and 100

## Data Availability

The original contributions presented in this study are included in the article. Further inquiries can be directed to the corresponding author.
